# Fosfomycin—Overcoming Problematic In Vitro Susceptibility Testing and Tricky Result Interpretation: Comparison of Three Fosfomycin Susceptibility Testing Methods

**DOI:** 10.3390/antibiotics13111049

**Published:** 2024-11-05

**Authors:** Jan Závora, Gabriela Kroneislová, Marie Kroneisl, Václava Adámková

**Affiliations:** 1 Clinical Microbiology and ATB Centre, General University Hospital, 128 08 Prague, Czech Republic; gabriela.kroneislova@vfn.cz (G.K.); vaclava.adamkova@vfn.cz (V.A.); 2 Department of Medical Microbiology, Palacky University, 779 00 Olomouc, Czech Republic; 3 Department of Clinical Pharmacy and Pharmacology, University Medical Center Groningen, University of Groningen, 9700 Groningen, The Netherlands; kroneisl.marie@gmail.com; 4 Department of Surgery, University Hospital Bulovka, 180 00 Prague, Czech Republic

**Keywords:** fosfomycin, difficult-to-treat, susceptibility, agar dilution, Etest, disc diffusion

## Abstract

**Background:** Fosfomycin (FOS) is an older antimicrobial agent newly rediscovered as a possible treatment for infections with limited therapeutic options (e.g., Gram-negative bacteria with difficult-to-treat resistance, DTR), especially in intravenous form. However, for correct usage of FOS, it is necessary to have a reliable susceptibility testing method suitable for routine practice and robust interpretation criteria. **Results:** The results were interpreted according to 2023 interpretation criteria provided by the European Committee on Antimicrobial Susceptibility Testing (EUCAST). DTR Gram-negatives were more likely to be resistant to FOS (45% in Enterobacterales and 20% in *P. aeruginosa*) than non-DTR (10% and 6.7%, resp.). All isolates of *S. aureus* were susceptible to FOS. In Gram-negatives, all agreement values were unacceptable. Etest^®^ performed better in the DTR cohort (categorical agreement, CA, 80%) than in the non-DTR cohort (CA 45.7%). There were no very major errors (VREs) observed in *P. aeruginosa*. *S. aureus* had surprisingly low essential agreement (EA) rates (53% for MRSA and 47% for MSSA) for Etest^®^, but categorical agreement was 100%. **Methods:** A total of 130 bacterial isolates were tested and compared using the disc diffusion method (DD) and gradient strip method (Etest^®^) with the reference method (agar dilution, AD). The spectrum of isolates tested was as follows: 40 Enterobacterales (20 DTR vs. 20 non-DTR), 30 *Pseudomonas aeruginosa* (15 DTR vs. 15 non-DTR), and 60 *Staphylococcus aureus* (30 methicillin-susceptible, MSSA, vs. 30 methicillin-resistant, MRSA). **Conclusions:** Neither one of the tested methods was identified as a suitable alternative to AD. It would be beneficial to define more interpretation criteria, at least in some instances.

## 1. Introduction

Fosfomycin (FOS) is a newly rediscovered antimicrobial agent first isolated in 1969 from bacterial cultures of *Streptomyces fradiae*, *S. viridochromogenes*, and *S. wedomorensis*. This molecule is a phosphoric acid derivate with very low molecular mass (138 Da), which contributes to good penetration into various compartments. A unique epoxide group in the molecule accounts for its bioavailability [1].

Fosfomycin can be administered in oral or intravenous form depending on the indication. The oral form of fosfomycin is indicated for the treatment of non-complicated urinary tract infections caused mainly by susceptible strains of *Escherichia coli* (including extended-spectrum beta-lactamase producers). Intravenous forms of fosfomycin are indicated in a range of infections including complicated urinary tract infections, infective endocarditis, bone and joint infections, hospital-acquired and ventilator-acquired pneumonia, complicated skin and soft tissue infections, bacterial meningitis, complicated intraabdominal infections, and bacteriaemia associated with the above-mentioned infections. Fosfomycin can be administered either as a monotherapy or in combination with other antibiotics [2,3].

The spectrum of activity of fosfomycin consists of aerobic Gram-positives (e.g., *Staphylococcus aureus* and some strains of *Streptococcus pneumoniae*), Gram-negatives (e.g., *E. coli*, *Citrobacter* sp., *Salmonella enterica*, some strains of *Klebsiella* sp., and *Pseudomonas aeruginosa*), and anaerobic bacteria (e.g., *Fusobacterium* sp. and *Peptostreptococcus* sp.) [2].

Clinical use of fosfomycin demands a reliable method for in vitro susceptibility testing. Neither the disc diffusion method nor the broth microdilution method provides truly correct results of fosfomycin susceptibility testing. Both the European Committee on Antimicrobial Susceptibility Testing (EUCAST) and the Clinical and Laboratory Standards Institute (CLSI) recommend agar dilution as a gold standard method for this purpose. Recently, the number of interpretation criteria (breakpoints) for fosfomycin has been largely reduced by the EUCAST, with both mentioned authorities providing breakpoints only for *E. coli* isolates originating from the urinary tract [4,5].

This study aims to compare three susceptibility testing methods (disc diffusion, gradient strip, and agar dilution) and to confirm the superiority of agar dilution on isolates of Enterobacterales, *P. aeruginosa*, and *S. aureus*. It is also important to discuss the great potential of fosfomycin as an alternative or synergic agent for infections caused by pathogens with limited treatment options. This opportunity is currently narrowed down by interpretation criteria not being available.

## 2. Results

In Gram-negative bacteria adhering to the DTR definition, the resistance to FOS was higher in both Enterobacterales and *P. aeruginosa* cohorts (40% and 20%, respectively) vs. non-DTR (10% and 6.7%). Similarly, the MIC_90_ of the DTR cohorts (128 mg/L and >256 mg/L, resp.) was also higher than that of the non-DTR (32 mg/L and 128 mg/L, resp.). All *S. aureus* isolates tested were susceptible to FOS (according to EUCAST FOS ECOFF). The results of fosfomycin susceptibility of all isolates in this study were interpreted according to the 2023 criteria provided by the EUCAST [4] and are summarized in Table 1 and Table 2.

### 2.1. Comparison of Methods for Gram-Negative Bacteria

The results in Gram-negatives were widely dispersed, and all calculated agreement values were unacceptable. Surprisingly, the gradient strips (Etest^®^) had better performance in the DTR cohort; categorical agreement (CA) was 80% for DTR Enterobacterales as well as *P. aeruginosa* vs. 40% and 53.3%, resp., in the non-DTR. However, the DTR cohort of Enterobacterales was the only one with very major errors (VMEs) detected for Etest^®^ (12.5%). See all values in Table 3, with the correlation of data in Table 4, Table 5 and Table 6.

In comparison with the reference method, the disc diffusion (DD) method produced VMEs in both Enterobacterales cohorts (12.5% in DTR and 100% in non-DTR). VMEs were not observed in *P. aeruginosa* cohorts at all, and better results were obtained with DD than Etest^®^ in the DTR *P. aeruginosa* cohort. Unacceptable major errors were calculated in all instances in Gram-negatives. Generally, CA was better for disc diffusion than gradient strip (e.g., DTR *P. aeruginosa* 80% for Etest^®^ and 93.3% for disc diffusion). 

Table 4 and Table 5 illustrate that in Gram-negatives, Etest^®^ gives generally higher MIC values, which pushes 30% (*n* = 12) Enterobacterales and 33% (*n* = 10) *P. aeruginosa* strains above the interpretation criteria (32 mg/L for Enterobacterales and 256 mg/L for *P. aeruginosa*) and makes them falsely resistant in comparison with the reference method. This was confirmed by a high percentage of difference bias, which was in Gram-negatives acceptable only in DTR Enterobacterales (+10%).

### 2.2. Comparison of Methods for S. aureus

**Figure 1:**
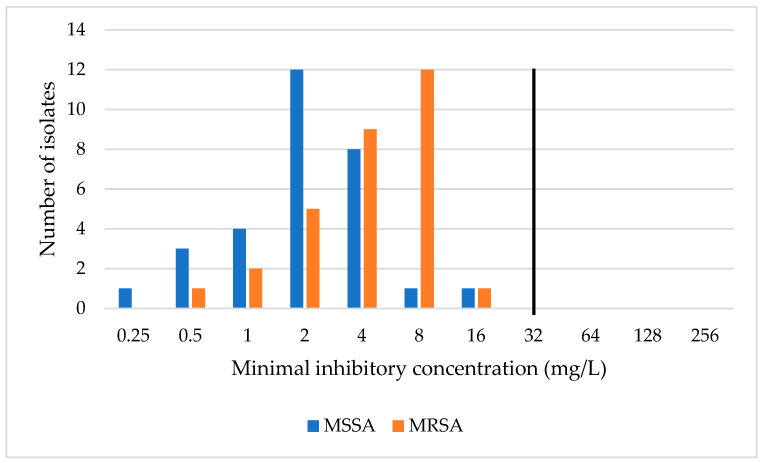
Fosfomycin MIC distribution—MSSA vs. MRSA; the black line illustrates an ECOFF.

The susceptible status of these isolates (*n* = 60) means that CA was 100% in both cohorts (MSSA and MRSA). However, essential agreement (EA) values were surprisingly low (53% for MRSA and 47% for MSSA) (Table 3). The MIC distribution of the MRSA cohort compared to the MSSA cohort (Figure 1) was slightly shifted to higher values. The MIC distributions in the comparison of the methods of both cohorts (Table 6) show that values determined using Etest^®^ were generally lower than with the reference method. This was then confirmed by calculating the difference bias, which, in both *S. aureus* cohorts (MSSA as well as MRSA), was unacceptably low (−56.6. and −66.6., resp.).

## 3. Discussion

In this study, three fosfomycin susceptibility testing methods were compared. Given the wide range of fosfomycin efficacy, Gram-negative (Enterobacterales and *P. aeruginosa*; differentiated in DTR and non-DTR cohorts) and Gram-positive (*S. aureus*, MSSA vs. MRSA) bacterial isolates were tested.

Fosfomycin (FOS) is a long-established antimicrobial agent that has gained attention more recently because of its potential to treat infections caused by bacteria with high-level resistance, such as DTR or MRSA isolates [6,7,8,9]. 

The strength of FOS lies in combination with other agents, especially beta-lactams [10]. Wu et al. showed that a combination of FOS with ceftazidime/avibactam (CAT) demonstrates a high level of synergy against carbapenem-resistant *Klebsiella pneumoniae*, most notably against metallo-beta-lactamase (MBL) producers (up to 64%). The synergistic effect of these two agents may be explained by their distinct mechanisms of action during cell wall synthesis [7]. Synergistic tendencies are detected even if there is resistance determined by one of the agents. Tüzemen et al. reported that CAT (susceptible) combined with FOS (resistant) displayed 42.3% synergy. Furthermore, in strains resistant to CAT and susceptible to FOS, the synergy was 66.7% [8]. 

Proper use of this agent in clinical practice requires a reliable and reproducible susceptibility testing method, correctly separating isolates with and without resistance.

The use of the most routine method, disc diffusion (DD), seems questionable. In the case of *E. coli* and *Citrobacter koseri*, the CA values in comparison with agar dilution (AD) were acceptable, but at the same time, there were high VME values (100% and 67%, resp.). This means that, when there is a resistant strain, DD mostly indicates susceptibility [11]. This occurs mainly because resistant isolates of these bacteria to fosfomycin are rare [10,12,13], making the number of resistant isolates needed to calculate VMEs very low. Further research regarding disc diffusion reliability when testing the fosfomycin-resistant isolates of *E. coli* would be beneficial.

Broth microdilution (BMD) is a method labeled as the gold standard for MIC determination in the majority of antibiotics [4]. As for fosfomycin, only a few authors have compared BMD with other methods given that the recommendations favor AD. According to recent studies, BMD generally shows higher MIC values than AD, leading to false resistance results, especially in non-*E. coli* isolates [9,14]. 

A commonly used MIC method in clinical practice is the gradient strip method (Etest^®^). Several authors report Etest^®^ displaying MIC values in Gram-negatives one or two dilutions lower than AD, especially in highly resistant strains like carbapenemase-producing Enterobacterales or *P. aeruginosa*. This leads to false results, which is significant because it can lead to treatment failure, consequently endangering the patient [9,15,16,17]. Given these findings, Etest^®^ may not be a suitable method of susceptibility determination for fosfomycin.

Some studies have compared automated methods such as BD Phoenix or BioMérieux VITEK to AD, showing similar results as Etest^®^ comparisons. Kowalska-Krochmal et al. used BD Phoenix, detecting the lowest error rates for *E. coli* (VMEs 9.09%) and *S. aureus* (VMEs 0%). On the other hand, *Enterobacter* sp. and *Klebsiella* spp. showed very high rates of VMEs, 53.83% and 38.37%, respectively [16]. Similarly, Massip et al. tested BioMérieux VITEK, and the MIC values were higher than AD, resulting in many false resistance results (ME 50% and 28% for *Enterobacter* sp. and *Klebsiella pneumoniae*, resp.) [11]. 

Generally, it seems that none of these methods can act as a suitable alternative to AD. Agar methods including disc diffusion and Etest^®^ produce many difficulties with the reading of results. Frequently, isolated colonies are found inside of the inhibition zones [18,19], which EUCAST recommends ignoring and CLSI recommends considering [4,5]. This inconsistency can lead to unreliable results. Furthermore, BMD is discouraged because of the “skipped wells” phenomenon occurring when there is no visible growth in a well before the MIC [14].

According to the above-mentioned studies, fosfomycin is confirmed to have a broad spectrum of action, including against highly resistant strains such as carbapenemase producers. Infections caused by these pathogens demand treatment options that are not available in some cases. I.v. fosfomycin could be a solution. Therefore, it is unfortunate, that in a new edition of EUCAST breakpoints tables (2024), breakpoints for fosfomycin were greatly reduced. Currently, there are breakpoints available only for *E. coli* isolates originating from the urinary tract. The one remaining MIC breakpoint was lowered this year from 32 mg/L to 8 mg/L, probably because all non-*E. coli* Enterobacterales were excluded [4]. Since other Enterobacterales were included in this study, the 2023 breakpoint of 32 mg/L was used to interpret the results. Recommendations by the CLSI are similarly affected. The future of fosfomycin breakpoints can lie in combination breakpoints because synergy is a real strength of FOS. One such combination interpretation criterion already exists: the CLSI provides an amikacin + FOS (ratio 5:2) breakpoint for aerosolized administration [5].

In our study, the disc diffusion method and gradient strip method were compared against the reference agar dilution method. In Gram-negative bacteria, neither one of the methods performed as a suitable alternative to AD for i.v. fosfomycin susceptibility testing. Low rates of categorical agreement were calculated across all of the cohorts of Gram-negative rods tested. This correlates with the results of several authors [16,17,20]. Routine methods like disc diffusion and Etest^®^ may be sufficient only for *E. coli*-caused infections of the lower urinary tract treated with the oral form of the agent [6,11].

Surprisingly, Etest^®^ performed better for isolates of Enterobacterales and *P. aeruginosa* with the difficult-to-treat type of resistance (DTR) than for non-DTR isolates. This may be caused by the higher number of resistant isolates in the DTR cohort with an MIC well above the breakpoint, which leads to better agreement of the methods. 

In several instances, DD performed better than Etest^®^, such as in the non-DTR Enterobacterales cohort (CA 70%) and the DTR *P. aeruginosa* cohort (CA 93.3%). However, these results were randomly distributed and the role of DD in intravenous fosfomycin susceptibility testing has yet to be assessed. 

In this study, *Acinetobacter* sp. isolates were not tested because there are no international or national guidelines available supporting the use of fosfomycin for infections caused by *Acinetobacter* sp. [21,22]. Nonetheless, several studies report the in vitro activity of fosfomycin against *Acinetobacter baumannii*, especially in combination with other agents providing the increase in bactericidal activity or prevention of antibiotic resistance [23]. In case of carbapenem-resistant *A. baumannii*, a combination of cefiderocol with fosfomycin performed better than colistin-based combinations [24]. 

Apart from Gram-negatives, FOS also seems to be a great therapeutic choice for Gram-positive pathogens. This study reported on in vitro fosfomycin activity toward *S. aureus*. Both the MSSA and MRSA cohort showed similar results: all isolates were susceptible, which means there were no errors. Regardless, essential agreement was low, around 50%, and MIC distribution shows lower MIC values measured by Etest^®^ supported by very low difference bias values (in both *S. aureus* cohorts, lower than −50%). This means that, if there were Fosfomycin-resistant strains included, there is a potential for false susceptibilty results, i.e., very major errors. This highlights the importance of further testing of Fosfomycin-resistant isolates.

Goer et al. reports better EA for *S. aureus* than in this study (92.9%), but the MIC values determined using Etest^®^ were also lower than agar dilution, at least by one two-fold dilution [15]. Similarly, in a previously published Polish study, *S. aureus* isolates display low essential agreement between Etest^®^ and AD (70%) with Etest^®^ showing lower values. The same study also reports on coagulase-negative staphylococci with a high rate of VMEs (21.43%) [16]. 

It has been reported that fosfomycin supposedly has a high level of synergy with Gram-positive spectrum antibiotics such as linezolid and daptomycin. These combinations could be beneficial for severe infections caused by highly resistant Gram-positive bacteria, including MRSA or vancomycin-resistant enterococci [25,26]. Enterococci are rarely resistant to fosfomycin [27]. 

As mentioned above, this study shows that neither one of the tested methods performed adequately compared to the reference method. AD represents a challenge given that it is usually a time-consuming and labour-intensive method. However, there are commercial kits available for fosfomycin including ready-to-use plates with wells filled with agar, requiring the laboratory personnel to only spot the bacterial suspension in each well. This makes the method more user friendly [28].

There are several limitations to this study. Firstly, the Enterobacterales cohort joins together *E. coli* and other Enterobacterales because of the low number of *E. coli* in the mix. This is because, generally, *E. coli* isolates with difficult-to-treat resistance are rare in the Czech Republic. In the case of fosfomycin, the results for *E. coli* may potentially be different than other Enterobacterales [16,29]. Secondly, no fosfomycin-resistant isolates of *S. aureus* were included in the study. This is because there were no resistant isolates detected in patient samples from our hospital. Thirdly, the isolates tested were collected in a single facility, which may have affected cohort diversity and thus the results. This also has an advantage: the authors have access to the strains and all data necessary from their facility and do not have to rely on data transfer.

## 4. Materials and Methods

### 4.1. Isolates

In this study, three fosfomycin susceptibility testing methods (agar dilution as a reference method, the disc diffusion method, and the gradient strip method) on 130 bacterial strains were compared. The spectrum of tested bacteria was as follows: isolates of Enterobacterales (*E. coli* (*n* = 4), *Klebsiella pneumoniae* (*n* = 24), *Enterobacter cloacae* complex (*n* = 12); total *n* = 40), *P. aeruginosa* isolates (*n* = 30), and *S. aureus* isolates (*n* = 60). Enterobacterales and *P. aeruginosa* cohorts include an equal number of strains with difficult-to-treat type of resistance (DTR) and an equal number of susceptible strains (non-DTR). Gram-negative isolates were classified as DTR if they were resistant to all first-line antibiotics: beta-lactams (penicillins, cephalosporins, and carbapenems) and fluoroquinolones [30]. The *S. aureus* cohort consists of an equal number of methicillin-susceptible and methicillin-resistant isolates (MSSA, resp. MRSA). The isolates were obtained from routinely examined clinically valid samples (e.g., blood culture, bronchoalveolar lavage, and abdominal fluid) from patients hospitalized in a university hospital with approx. 1500 beds.

### 4.2. Susceptibility Testing

#### 4.2.1. Agar Dilution—Reference Method

A bacterial suspension adjusted to 0.5 McFarland turbidity (McF) was first diluted 10 times using 0.9% NaCl solution. 2 µL of the final suspension was spotted into the wells of the agar dilution plates of the commercial kit AD Fosfomycin 0.25–256 (Liofilchem, Roseto degli Abruzzi, Italy), which were then incubated at 37 °C for 24 h. Minimum inhibitory concentration (MIC) values were recorded as the lowest concentration of fosfomycin needed to inhibit growth completely. 

#### 4.2.2. Gradient Strips

Overnight bacterial growth was suspended in saline solution, adjusted to 0.5 McF, and spread on Mueller–Hinton (MH) agar for Etest^®^ (MHE agar; bioMérieux SA, Marcy l’Etoile, France). Gradient strips (Etest^®^; bioMérieux SA, Marcy-l’Etoile, France) saturated with FOS were applied to the plate and incubated in 37 °C for 24 h.

#### 4.2.3. Disc Diffusion

A bacterial suspension adjusted to 0.5 McF was spread on MH agar and FOS antibiotic discs (200 µg) were applied to each plate in duplicate (MH agar and discs: OXOID/Thermo Scientific, Waltham, MA, USA). The MH plates were then incubated in 37 °C for 24 h.

#### 4.2.4. Quality Control

To confirm the methodology, the following strains were used as controls: *Escherichia coli* ATCC 25922, *Pseudomonas aeruginosa* ATCC 27853, and *Staphylococcus aureus* ATCC 29213. These control strains were tested in parallel with test isolates for each susceptibility test.

#### 4.2.5. Note on the Interpretation of the Results

Interpretation criteria for i.v. fosfomycin are currently available only for *E. coli* strains originating from the urinary tract (EUCAST, CLSI, 2024) [4,5]. Therefore, the susceptibility results of the remaining Enterobacterales were interpreted according to EUCAST 2023 breakpoints for i.v. fosfomycin. Because there has never been a fosfomycin breakpoint for *P. aeruginosa,* susceptibility was determined using the epidemiological cutoff (ECOFF) of 256 mg/L, which was provided by the EUCAST. The breakpoint for *S. aureus* was also discontinued in 2024, but the same value is still published as an ECOFF (32 mg/L) by the EUCAST [4]. 

### 4.3. Study Design

All three methods were performed at the same time and the results were read by two professionals; the resulting values were calculated as an arithmetic mean. The categories susceptible and resistant were established according to EUCAST breakpoints if available. 

As recommended by ISO 20776-2:2021 [31], the methods were compared using essential and categorical agreement rates and error rates. Essential agreement was defined as the concordance of the tested methods within ±1 log2 dilution compared to the reference method and discordant when it was within ±2 log2 dilution or more. Categorical agreement was defined as the results falling within the same category of susceptibility. The category errors were determined as very major errors or major errors. Very major errors (VMEs) occurred when the reference methods showed the isolate as resistant and the tested method as susceptible; major errors (MEs) represented an isolate deemed susceptible by the reference method but resistant by the tested method. There were no minor errors because fosfomycin does not have an intermediate susceptibility category according to the EUCAST [4]. For better comparison, difference bias was also calculated. Errors were unacceptable if the value was ≥1.5% for very major errors and ≥3.0% for major errors, and bias was unacceptable if ≥±30% [31].

Furthermore, agar dilution and Etest^®^ were also compared by analyzing the MIC distribution and calculating MIC_50_ and MIC_90_ (the lowest concentration of an antimicrobial agent inhibiting 50% and 90% of isolates, respectively, in a microbial population) of these methods.

## 5. Conclusions

Intravenous fosfomycin seems to be a suitable antimicrobial agent for the treatment of infections caused by Gram-negative and Gram-positive bacteria, including highly resistant strains. However, in routine practice, it is difficult to determine susceptibility reliably. The data presented in this study did not identify any suitable alternative method to agar dilution, which confirms the validity of the CLSI and EUCAST recommendations. There is still a need for the testing of fosfomycin-resistant strains of *E. coli* and *S. aureus*. With commercially available agar dilution plates, the only reliable method (the reference method) became more attractive for routine laboratory testing. 

## Figures and Tables

**Table 1:** MIC distributions and percentage of resistance to fosfomycin for Gram-negative rods.

	Enterobacterales			*Pseudomonas aeruginosa*		
	non-DTR (*n* = 20)	DTR (*n* = 20)	Total (*n* = 40)	non-DTR (*n* = 15)	DTR (*n* = 15)	Total (*n* = 30)
MIC_50_ (mg/L)	16	32	16	64	32	32
MIC_90_ (mg/L)	32	128	128	128	>256	>256
Range (mg/L)	0.5 to 64	0.5 to 256	0.5 to 256	4 to >256	4 to >256	4 to >256
% resistance (*n*)	10 (2)	40 (8)	25 (10)	6.7 (1)	20 (3)	13.3 (4)

DTR (difficult-to-treat resistance) cohort: resistant to all beta-lactams, incl. carbapenems, and fluoroquinolones; non-DTR cohort: susceptible to all of the above-mentioned antibiotics. Susceptibility interpretation criteria: Enterobacterales, EUCAST 2023 breakpoint for i.v. fosfomycin = 32 mg/L; *P. aeruginosa*, EUCAST epidemiological cutoff = 256 mg/L.

**Table 2:** MIC distributions and percentage of resistance to fosfomycin for Staphylococcus aureus.

	MSSA (*n* = 30)	MRSA (*n* = 30)	Total (*n* = 60)
MIC_50_ (mg/L)	2	4	4
MIC_90_ (mg/L)	4	8	8
range (mg/L)	0.25–16	0.5–16	0.25–16
% resistance (*n*)	0	0	0

MSSA, methicillin-susceptible *S. aureus*; MRSA, methicillin-resistant *S. aureus*. Susceptibility interpretation criteria: EUCAST epidemiological cutoff = 32 mg/L.

**Table 3:** Essential agreement, categorical agreement, error rates, and bias of clinical isolates of DTR and non-DTR Gram-negative rods (Enterobacterales and *P. aeruginosa*) and Staphylococcus aureus (MRSA and MSSA).

Fosfomycin, Reference Method AD	Agreement Values [%]		Error Values [%(*n*)]		Bias [%]
	EA	CA	VME	ME	
**DTR Gram-negatives (*n* = 35)**					
Etest^®^	60	80	9 (1)	25 (6)	+40
DD	NA	85.7	9 (1)	16.7 (4)	NA
**non-DTR Gram-negatives (*n* = 35)**					
Etest^®^	34.3	45.7	0	50 (16)	+77.1
DD	NA	60	66.7 (2)	37.5 (12)	NA
**DTR Enterobacterales (*n* = 20)**					
Etest^®^	75	80	12.5 (1)	25 (3)	+10
DD	NA	80	12.5 (1)	25 (3)	NA
**non-DTR Enterobacterales (*n* = 20)**					
Etest^®^	50	40	0	55.6 (10)	+85
DD	NA	70	100 (2)	22.2 (4)	NA
**DTR *P. aeruginosa* (*n* = 15)**					
Etest^®^	40	80	0	25 (3)	+80
DD	NA	93.3	0	8.3 (1)	NA
**non-DTR *P. aeruginosa* (*n* = 15)**					
Etest^®^	53.3	53.3	0	42.9 (6)	+66.6
DD	NA	46.6	0	57.1 (8)	NA
***S. aureus* methicillin-resistant, MRSA (*n* = 30)**					
Etest^®^	53	100	0	0	−66.6
***S. aureus*** **methicillin-susceptible, MSSA (*n* = 30)**					
Etest^®^	47	100	0	0	−56.6

AD, agar dilution; CA, categorical agreement; DD, disk diffusion; DTR, difficult-to-treat; EA, essential agreement; ME, major error; NA, not applicable; VME, very major error. Data for disc diffusion in *S. aureus* are not included. There are no breakpoints for interpretation. Bias was calculated as a difference bias, acceptable ±30%.

**Table 4:** Agar dilution compared to Etest^®^ (MIC vs. MIC) and disc diffusion (MIC vs. disc diameter) for Enterobacterales.

**ENT**		**AD FOS MIC (mg/L)**												
		**0.125**	**0.25**	**0.5**	**1**	**2**	**4**	**8**	**16**	**32**	**64**	**128**	**256**	**>256**
Etest® FOS MIC (mg/L)	**0.125**													
	**0.25**													
	**0.5**			2										
	**1**								1					
	**2**							1						
	**4**													
	**8**			2			2	1						
	**16**								2					
	**32**								2 + 1	3		1		
	**64**							2	4	2 + 3	1	1		
	**128**										1	3		
	**256**								2		2		1	
	**>256**													
**ENT**		**AD FOS MIC (mg/L)**												
		**0.125**	**0.25**	**0.5**	**1**	**2**	**4**	**8**	**16**	**32**	**64**	**128**	**256**	**>256**
DD FOS (mm)	**6**												1	
	**7**													
	**8**													
	**9**													
	**10**													
	**11**													
	**12**													
	**13**													
	**14**													
	**15**											2		
	**16**										1			
	**17**										1	1		
	**18**								2					
	**19**											1		
	**20**									2 + 3				
	**21**							1 + 1	3 + 1	1	1			
	**22**								1	1				
	**23**						2		2		1	1		
	**24**							1	1	1				
	**25**								1					
	**26**													
	**27**			1										
	**28**							1						
	**29**								1					
	**30**			1 + 2										

AD, agar dilution; DD, disc diffusion; DTR, difficult-to-treat; ENT, Enterobacterales; FOS, fosfomycin. Bold black lines illustrate breakpoints/ECOFF. Black numbers = non-DTR cohort, red numbers = DTR cohort. Green fields illustrate essential agreement.

**Table 5:** Agar dilution compared to Etest^®^ (MIC vs. MIC) and disc diffusion (MIC vs. disc diameter) for Pseudomonas aeruginosa.

**PSAE**		**AD FOS MIC (mg/L)**												
		**0.125**	**0.25**	**0.5**	**1**	**2**	**4**	**8**	**16**	**32**	**64**	**128**	**256**	**>256**
Etest^®^ FOS MIC (mg/L)	**0.125**													
	**0.25**													
	**0.5**													
	**1**													
	**2**						1							
	**4**													
	**8**						1	1						
	**16**								1					
	**32**								1					
	**64**							1		2 + 1				
	**128**								1	1 + 4	1			
	**256**										1			
	**>256**									2	5 + 1	1		1 + 3
**PSAE**		**AD FOS MIC (mg/L)**												
		**0.125**	**0.25**	**0.5**	**1**	**2**	**4**	**8**	**16**	**32**	**64**	**128**	**256**	**>256**
DD FOS (mm)	**6**													1 + 3
	**7**													
	**8**													
	**9**													
	**10**													
	**11**													
	**12**										1			
	**13**													
	**14**													
	**15**													
	**16**										1	1		
	**17**										1			
	**18**										1			
	**19**										1			
	**20**									1	2			
	**21**									1 + 2				
	**22**								1	1 + 4	1			
	**23**							1						
	**24**													
	**25**									1				
	**26**													
	**27**													
	**28**								1					
	**29**													
	**30**						1 + 1	1	1					

AD, agar dilution; DD, disc diffusion; DTR, difficult-to-treat; FOS, fosfomycin; PSAE, *P. aeruginosa*. Bold black lines illustrate breakpoints/ECOFF. Black numbers = non-DTR cohort, red numbers = DTR cohort. Green fields illustrate essential agreement.

**Table 6:** Agar dilution compared to Etest^®^ (MIC vs. MIC) and disc diffusion (MIC vs. disc diameter) for Staphylococcus aureus.

**STAU**		**AD FOS MIC (mg/L)**												
		**0.125**	**0.25**	**0.5**	**1**	**2**	**4**	**8**	**16**	**32**	**64**	**128**	**256**	**>256**
Etest^®^ FOS MIC (mg/L)	**0.125**			1	1	2								
	**0.25**			1	2 + 1	2 + 3								
	**0.5**		1	1 + 1	1 + 1	2 + 2	1							
	**1**					1	4 + 2	1						
	**2**					4	2 + 3	5						
	**4**					1	2 + 3	2	1					
	**8**							1 + 2						
	**16**							2	1					
	**32**													
	**64**													
	**128**													
	**256**													
	**>256**													
**STAU**		**AD FOS MIC (mg/L)**												
		**0.125**	**0.25**	**0.5**	**1**	**2**	**4**	**8**	**16**	**32**	**64**	**128**	**256**	**>256**
DD FOS (mm)	**6**													
	**7**													
	**8**													
	**9**													
	**10**													
	**11**													
	**12**													
	**13**													
	**14**													
	**15**													
	**16**							1						
	**17**													
	**18**						1							
	**19**													
	**20**			1		1	3	1 + 4	1					
	**21**					1 + 2	1 + 1	3						
	**22**					2 + 2	2 + 3	3						
	**23**							1						
	**24**			1	1	2	1							
	**25**				1 + 1	2	2 + 1							
	**26**				3	3	2							
	**27**			2		1								
	**28**													
	**29**					1								
	**30**			1					1					

AD, agar dilution; DD, disc diffusion; FOS, fosfomycin; STAU, *S. aureus*. Bold black lines illustrate breakpoints/ECOFF. There are no DD interpretation criteria for *S. aureus*. Black numbers = MSSA cohort; red numbers = MRSA cohort. Green fields illustrate essential agreement.

## Data Availability

The original contributions presented in the study are included in the article. Further inquiries can be directed to the corresponding author.
